# The efficacy of chimeric antigen receptor (CAR) immunotherapy in animal models for solid tumors: A systematic review and meta-analysis

**DOI:** 10.1371/journal.pone.0187902

**Published:** 2017-11-15

**Authors:** Yingcheng Wu, Ran Xu, Keren Jia, Hui Shi

**Affiliations:** 1 Medical School of Nantong University, Jiangsu, China; 2 Department of Thoracic Surgery, Affiliated Hospital of Nantong University, Nantong, Jiangsu, China; Duke University School of Medicine, UNITED STATES

## Abstract

**Background:**

Most recently, an emerging theme in the field of tumor immunology predominates: chimeric antigen receptor (CAR) therapy in treating solid tumors. The number of related preclinical trials was surging. However, an evaluation of the effects of preclinical studies remained absent. Hence, a meta-analysis was conducted on the efficacy of CAR in animal models for solid tumors.

**Methods:**

The authors searched PubMed/Medline, Embase, and Google scholar up to April 2017. HR for survival was extracted based on the survival curve. The authors used fixed effect models to combine the results of all the trials. Heterogeneity was assessed by I-square statistic. Quality assessment was conducted following the Stroke Therapy Academic Industry Roundtable standard. Publication bias was assessed using Egger's test.

**Results:**

Eleven trials were included, including 54 experiments with a total of 362 animals involved. CAR immunotherapy significantly improved the survival of animals (HR: 0.25, 95% CI: 0.13–0.37, P < 0.001). The quality assessment revealed that no study reported whether allocation concealment and blinded outcome assessment were conducted, and only five studies implemented randomization.

**Conclusions:**

This meta-analysis indicated that CAR therapy may be a potential clinical strategy in treating solid tumors.

## Introduction

Cancer is one of the leading causes of death around the world[1]. However, the leaps and bounds of chimeric antigen receptor (CAR) immunotherapy is changing such a situation. CARs can target specific antigen of tumor cells, therefore activating T cells and inducing robust antitumor effects. In the field of hematologic malignancies, the surging of CAR immunotherapy has demonstrated remarkable success[2].

Compared with hematologic malignancies, solid tumors remained a significant challenge to CAR-T immunotherapy. So far, an increasing number of preclinical trials have focused on solid tumors, targeting at carcinoembryonic antigen (CEA), interleukin 13 receptor (IL-13R), human epidermal growth factor receptor 2 (HER2), fibroblast activation protein (FAP) and so on. At present, more and more scientists are devoted to searching for potential targets.

Most recently, publications on preclinical trials of solid tumors have abounded, and relevant phase I or phase I/II clinical trials have just been initiated[3]. However, an evaluation of the effects of preclinical studies remains absent. Which target of CARs will induce better or worse outcomes? What is the role of CARs in treating different types of cancer? Are the outcomes reliable in preclinical studies? These questions still remain unknown.

Here, we conducted a meta-analysis of animal models in order to evaluate the potential value of CAR-T therapy for solid tumors based on the preclinical trials. Also, we attempted to explore the experimental design features of current studies in order to point out the possible shortcomings of the preclinical experimental designs and the future clinical trials.

## Materials and methods

### Literature search

We search trials among PubMed/Medline, Embase, and Google scholar up to April 2017. Key words included "chimeric antigen receptor", "CAR", "solid tumor", "GBM", "lung cancer", "colorectal cancer", "pancreatic cancer", "prostate cancer", "ovarian cancer", "breast cancer", "preclinical". All additional studies of potential interest were retrieved for further analysis. All publications were written in English.

All the related publications were screened independently by two reviewers (YW and RX) to identify studies that met the inclusion criteria (below).

### Inclusion and exclusion criteria

Eligible studies should meet these standards. (1) Participant: the trials be conducted in animal models. (2) Intervention: CAR immunotherapy. (3) Control: the researchers should make at least one comparison between CAR T cell group and NT T cell group or untreated group. (4) Outcome: the survival curve should be reported. For trials that compared CAR and control group in more than one tumor model, the survival curve of each tumor model was included. If there was a disagreement between the two reviews, another reviewer (HS) reviewed it and a final consensus was reached.

### Data extraction

Three reviewers (YW, RX, and KJ) independently extracted data with a extraction form, and we checked all the data very carefully. We identified all the studies with the first author and the year of publication. We extracted the following information from the reports: first-author; year of publication; animal species; age; experimental group; control group; animal number; type of model; target; the generation of CAR; the type of cancer; the Kaplan-Meier survival curve. When the data was reported merely in image format, we attempted to contact the correspondence author of the publication to ask for the original data. If there was no reply or no useful information, Engauge Digitizer software V9.7 for macOS 10.12.3 was used to measure graphically the data as presented. When different CAR T cells were evaluated in multiple groups in one publication, the data in each group were extracted as an individual experiment for analysis. If the efficacy of different doses of CAR T cells were evaluated, all the valid hazard ratios for survival would be extracted.

### Quality assessment

A latest 2009 version of the initial Stroke Therapy Academic Industry Roundtable (STAIR) standard was applied to assess the quality of the studies[4]. It includes: (1) sample-size calculation; (2) inclusion and exclusion criteria; (3) randomization; (4) allocation concealment; (5) reporting of animals excluded from analysis; (6) blinded assessment of outcome; (7) reporting potential conflicts of interest and study funding. Three reviews (YW, RX, and KJ) assessed the qualities in all included studies and presented as a "yes" or "no". The "unclear" means the quality was not clear. The image was made with Numbers V4.1 software.

### Data analysis

Statistical analysis, forest plots and detection of publication bias were carried out with Stata SE 14.1 for macOS 10.12.3 (StataCorp, College Station, TX, USA). The data of survival was extracted by Engauge Digitizer software V9.7 for macOS 10.12.3. The ln(HR) value and se(ln(HR)) value were calculated based on an Excel spreadsheet developed by Matthew Sydes and Jayne Tierney of the MRC Clinical Trials Unit, London, the United Kingdom[5, 6]. *P*≤0.05 was used to indicate a statistical significance. Heterogeneity was considered low, moderate or high for I- squared values <25%, 25–50% and >50%[7]. A fixed effect model would be used if the heterogeneity was low or moderate. If the heterogeneity was high, the analysis would be performed with a random effects model. Publication bias was assessed by Egger's test. If the p value is more than 0.1 in the Egger's test, it was considered insignificant for publication bias[8].

## Results

### Literature selection and study characteristics

The preliminary literature search included 3,199 relevant publications (S1 Fig). Of these, 3,157 studies contained commentaries, editorials, study protocols, and irrelevant themes. And they were excluded afterwards based on their titles or abstracts. The remaining 32 studies were reviewed in full text. After removing duplicated literatures, literatures without usable data and some ineligible literatures, we identified articles eligible for further review by screening texts. We identified fifteen trials including 54 experiments with a total of 362 animals involved[9–23]. The whole research process can be seen in the S1 Fig. All the studies reported the survival curve. The characteristics among these studies varied considerably. Main characteristics of those trials are available in the Table 1.

**Table 1:** Characteristics of the included animal studies.

First author	Year	Cancer	Target	n	CAR generation	Animals	immunocompetent / immunocompromised	Exp	Ctrl	model
**Choi**	2013	GBM	EGFRvIII	5/5/5/5	3	5-6-week-old NSG female mice	immunocompromised	5×10^5 EGFRvIII CAR T cells; 5×10^4 EGFRvIII CAR T cells; 5×10^3 EGFRvIII CAR T cells;	5×10^5 untreated CAR T cells	Intracranial glioma xenograft
**Ohno**	2013	GBM	EGFRvIII	10/5	3	5-6-week-old NOG female mice	immunocompromised	5×10^4 U87-EGFRvIII-Luc cells	5×10^4 mock-transduced T-cells	mice bearing human GBM xenografts
**Chow**	2013	GBM	EphA2	12/8/9	2	8-12-week-old ICR-SCID male mice	immunocompromised	1×10^6 EphA2 CAR T cells	1×10^6 NT T cells; untreated	Orthotopic xenograft SCID mouse model
**Kong**	2012	GBM	IL13R	13/12/4	2	6-8-week-old female nude rats	immunocompetent	5×10^6 IL13Rα2 CAR T cells	5×10^6 NT T cells; tumor only	a human glioma xenograft model
**Krebs**	2014	GBM	IL13R	11/11/11/11/9/10	2	ICR-SCID mice	immunocompromised	2×10^6 IL13KR CAR T cells; 2×10^6 IL13K CAR T cells; 2×10^6 IL13YR CAR T cells; 2×10^6 IL13Y CAR T cells;	2×10^6 NT T cells; untreated	an orthotopic xenograft SCID mouse model of GBM
**Zhou**	2013	lung cancer	EGFR	5/5	2	5-6-week-old NSG CB-17 mice	immunocompromised	2×10^6 EGFR CAR T cells	2×10^6 mock T cell	a xenogeneic model of advanced lung metastatic A549 cancer; an A431 tumorigenicity model; an A2780 s.c. tumor model
**Kakarla**	2013	lung cancer	FAP	8/9/9	2	8-12-week-old ICR-SCID male mice	immunocompromised	10×10^6 FAP CAR T cells	10×10^6 NT T cells; untreated	a human A549 lung cancer xenograft model; a loco-regional tumor model
**Ahmed**	2009	lung cancer	HER2	10/9/5/5	1	9-12-week-old ICR-SCID male mice	immunocompromised	10×10^6 HER2 CAR T cells(treated day 2); 10×10^6 HER2 CAR T cells(treated day 8);	10×10^6 NT T cells; Tumor	LM7 xenogeneic lung metastases model; a xenogeneic SCID mouse model
**MALIAR**	2012	pancreatic cancer	HER2	7/7	1	ICR-SCID male mice	immunocompromised	1×10^7 HER2 CAR T cells	1×10^7 CD24 CAR T cells	PAC Wapac-4 and Wapac-5 xenograft models
**Blat**	2014	colorectal cancer	CEA	7/7/7/7/7	2	CEABAC-2 and CEABAC-10 mice	immunocompetent	0.75×10^6 CEA CAR T cells; 1.5×10^6 CEA CAR T cells	0.75×10^6 irrelevant CAR T cells; 1.5×10^6 irrelevant CAR T cells; untreated	T-cell-transfer colitis and azoxymethane–dextran sodium sulfate model for colitis-associated colorectal cancer
**Zhu**	2015	GBM	CD133	7/7	3	6–8-week-old male NMRI nude mice	immunocompromised	2×10^6 CD133 CAR T cells	2×10^6 NT T cells	an orthotopic mouse model of GBM
**Slaney**	2016	breast cancer	HER2	7/7	2	8–12-week-old C57BL/6 gender-matched mice	immunocompetent	1×10^7 HER2 CAR T cells	untreated	an immunocompetent, self-antigen preclinical mouse model of orthotopic breast cancer
**Wu**	2015	ovarian cancer	B7H6	23/23	2	7–12-week-old C57BL/6 mice	immunocompetent	5×10^6 B7H6 CAR T cells	5×10^6 mock HER2 CAR T cells	a systemic T cell lymphoma model; an ovarian cancer model
**Shiina**	2016	GBM	PDPN	12/14/14	3	5–6-week-old NOG female mice	immunocompromised	2×10^6 NZ-1 CAR T cells	2×10^6 mock-transduced PBMCs	an intracranial glioma xenograft model
**Hong**	2016	ovarian cancer	L1-CAM	6/6/6/6	2	8-week-old NSG female mice	immunocompromised	5×10^6 L1-CAM CAR T cells	5×10^6 CD19 CAR T cells;5×10^6 mock-transduced T cells; PBS	a xenograft mouse model of ovarian cancer

Abbreviations: NOD, nonobese diabetic; SCID, severe combined immunodeficient; NT, nontransduced; SCID, severe combined immunodeficiency; s.c., subcutaneous; GBM, Glioblastoma; PBL, peripheral blood lymphocytes; PBMC, peripheral blood mononuclear cells; ICR, inverted cytokine receptor; 1G, first generation; 2G, second generation; PBS, phosphate buffer saline.

### Meta-analyses

**Fig 1:**
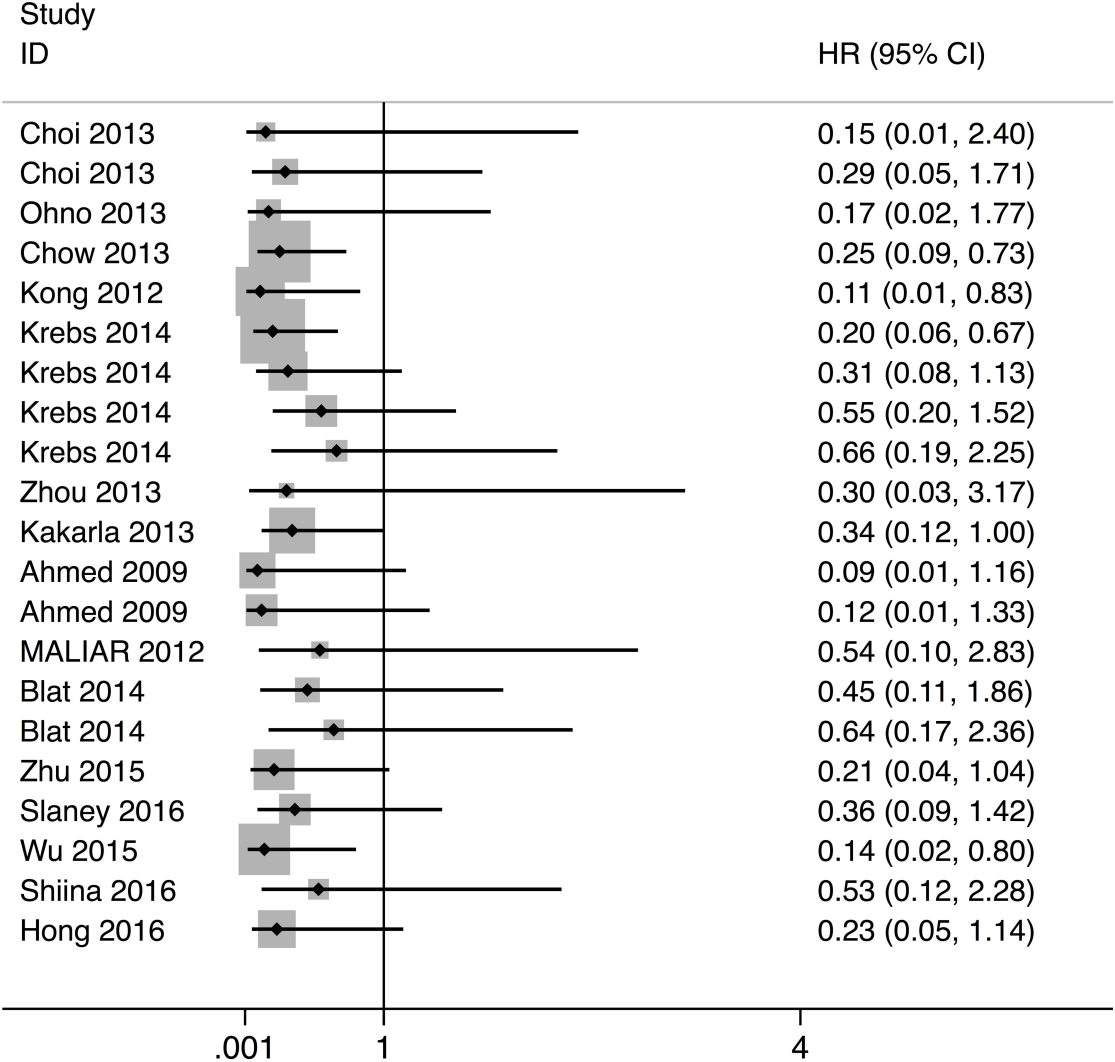
Forest plot of the meta-analysis for the hazard ratio.

The meta-analysis on survival time indicated that CAR immunotherapy was associated with a significantly prolonged survival (HR: 0.25, 95% CI: 0.13–0.37, P < 0.001) (Fig 1). And the heterogeneity was low (I-squared = 0.0%). We then conducted a subgroup analyses of year of publication, generation of CAR, type of cancer, type of animal model, and target (Table 2). The subgroup analysis manifested that, among all types of cancers, CAR immunotherapy was most efficient in ovarian cancer animals (HR: 0.170, 95% CI: -0.147–0.488). The subgroup analysis by target showed that HER2-CAR-T therapy is most efficient (HR: 0.203, 95% CI: -0.148–0.554). Also, a comparison between immunocompromised and immunocompetent animal models was also performed. Notably, no significant difference was observed between immunocompromised and immunocompetent animals (P = 0.712). This finding could be due to the lack of statistical power.

**Table 2:** Subgroup analysis by cancer type, target, generation, animal model, and publication year.

Cancer type	HR	95% CI
GBM	0.247	0.092–0.402
lung cancer	0.223	-0.080–0.526
colorectal cancer	0.524	-0.160–1.208
ovarian cancer	0.17	-0.147–0.488
**Target**		
EGFRvIII	0.216	-0.321–0.754
IL13	0.247	0.041–0.453
HER2	0.203	-0.148–0.554
CEA	0.524	-0.160–1.208
**Generation**		
1	0.143	-0.270–0.556
2	0.258	0.121–0.396
3	0.246	-0.101–0.592
**Animal model**		
immunocompromised	0.26	0.119–0.401
immunocompetent	0.207	-0.036–0.450
**Publication year**		
2009	0.103	-0.331–0.536
2012	0.146	-0.247–0.538
2013	0.270	0.039–0.500
2014	0.318	0.093–0.543
2015	0.166	-0.141–0.474
2016	0.315	-0.078–0.708

Subgroup analyses of less than two experiments were not performed due to the small sample size.

### Quality assessments and risk of bias

**Fig 2:**
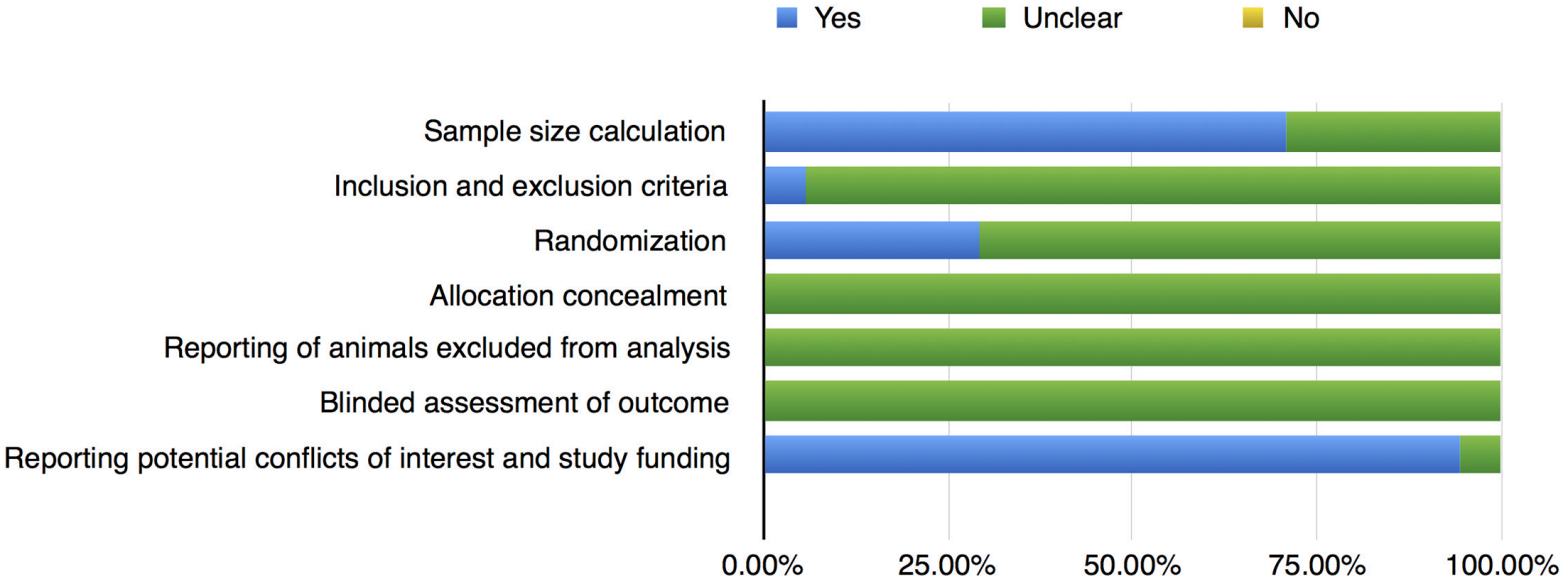
STAIR's risk of bias: Yes = low risk of bias, No = high risk bias, Unclear = unclear risk of bias.

The quality of the seventeen studies was assessed by the STAIR tool (Fig 2 and Table 3). According to the Egger's test, the P value was 0.013, which manifests that the publication bias did not exist.

**Table 3:** Quality assessment of the included trials.

Study	Sample size calculation	Inclusion and exclusion criteria	Randomization	Allocation concealment	reporting of animals excluded from analysis	blinded assessment of outcome	reporting potential conflicts of interest and study funding
**Choi 2013**	Unclear	Unclear	Unclear	Unclear	Unclear	Unclear	Yes
**Ohno 2013**	Yes	Unclear	Unclear	Unclear	Unclear	Unclear	Yes
**Chow 2013**	Yes	Unclear	Unclear	Unclear	Unclear	Unclear	Yes
**Kong 2012**	Yes	Unclear	Yes	Unclear	Unclear	Unclear	Yes
**Krebs 2014**	Yes	Unclear	Unclear	Unclear	Unclear	Unclear	Yes
**Zhou 2013**	Unclear	Unclear	Yes	Unclear	Unclear	Unclear	Yes
**Kakarla 2013**	Yes	Yes	Unclear	Unclear	Unclear	Unclear	Yes
**Ahmed 2009**	Yes	Unclear	Unclear	Unclear	Unclear	Unclear	Yes
**MALIAR 2012**	Yes	Unclear	Unclear	Unclear	Unclear	Unclear	Yes
**Blat 2014**	Unclear	Unclear	Unclear	Unclear	Unclear	Unclear	Yes
**Zhu 2015**	Yes	Unclear	Unclear	Unclear	Unclear	Unclear	Unclear
**Slaney 2016**	Yes	Unclear	Yes	Unclear	Unclear	Unclear	Yes
**Wu 2015**	Yes	Unclear	Unclear	Unclear	Unclear	Unclear	Yes
**Shiina 2016**	Yes	Unclear	Yes	Unclear	Unclear	Unclear	Yes
**Hong 2016**	Unclear	Unclear	Unclear	Unclear	Unclear	Unclear	Yes

This meta-analysis revealed that many common practices including randomization were not implemented in most of the trials. None of the published studies reported whether blinded assessment of outcome was carried out. Whether there existed any expectations or personal preferences was unclear. This made it difficult to find out that some outcome of experiments was in fact invalid.

## Discussion

To the best of our knowledge, this is the first meta-analysis which assessed efficacy of chimeric antigen receptor (CAR) immunotherapy in animal models for solid tumors. Publications on preclinical trials of solid tumors have abounded recently. Also, phase I and phase I/II clinical trials of CAR on solid tumors have just been initiated. The aim of this study is to assess the potential value of CAR-T therapy for solid tumors based on the preclinical trials.

### Main findings

Based on our analyses, CAR-T immunotherapy proved to generate a robust antitumor efficacy in animal models. The quality assessment manifested that there were some defects in the field of CAR preclinical research. No trials reported whether blinded outcome assessment or allocation concealment was performed. Only five studies implemented randomization, which may have induced uncertainties.

Our subgroup analysis illustrated that CAR immunotherapy was most efficient in ovarian cancer animals, and HER2-CAR-T cell therapy was demonstrated to be more effective. Inserestingly, between immunocompromised and immunocompetent animals models, no significant difference of efficacy was observed. This finding could be due to a lack of statistical power.

### Agreement/disagreement with previous study

To date, there is no meta-analysis evaluating CAR immunotherapy in animal models. A meta-analysis tended to evaluate the efficiency of CD19 CAR T cells for treatment of B cell malignancies [24]. Base on results of that meta-analysis, the number of CD19-CAR T cells have positive correlations with the clinical efficiency. Also, a systematic review had a discussion about the the increasing number of CAR trials[25].

### Limitations

This study does have some limitations. Firstly, all the preclinical trials evaluating CARs have comparatively small group sizes, leading to some uncertainties of outcomes. Secondly, doses of CAR-T cells varies in different experiments, ranging from 5×10^3 to 1×10^7. Therefore, some of the comparisons between CARs and control groups may be invalid, although we have excluded the invalid comparisons according to our criteria. Thirdly, we used Engauge Digitizer software in order to extract data from the survival curve. Minor distortion of effect sizes were likely to occurred. Fourth, the meta-analysis didn't directly address some elements, including duration of trial or selection of model.

## Conclusions

CAR immunotherapy appeared to inhibit the growth of solid tumors in animal models. CAR therapy may be a potential clinical strategy in treating solid tumors.

## Supporting information

S1 Fig The process of trial selection. (PDF)
