# Supplementary figures and images for: The efficacy of chimeric antigen receptor (CAR) immunotherapy in animal models for solid tumors: A systematic review and meta-analysis
Source: PLoS One. 2017 Nov 15;12(11):e0187902. doi: 10.1371/journal.pone.0187902 (PMC5687736; doi:10.1371/journal.pone.0187902)

**Fig S1.** The process of trial selection.

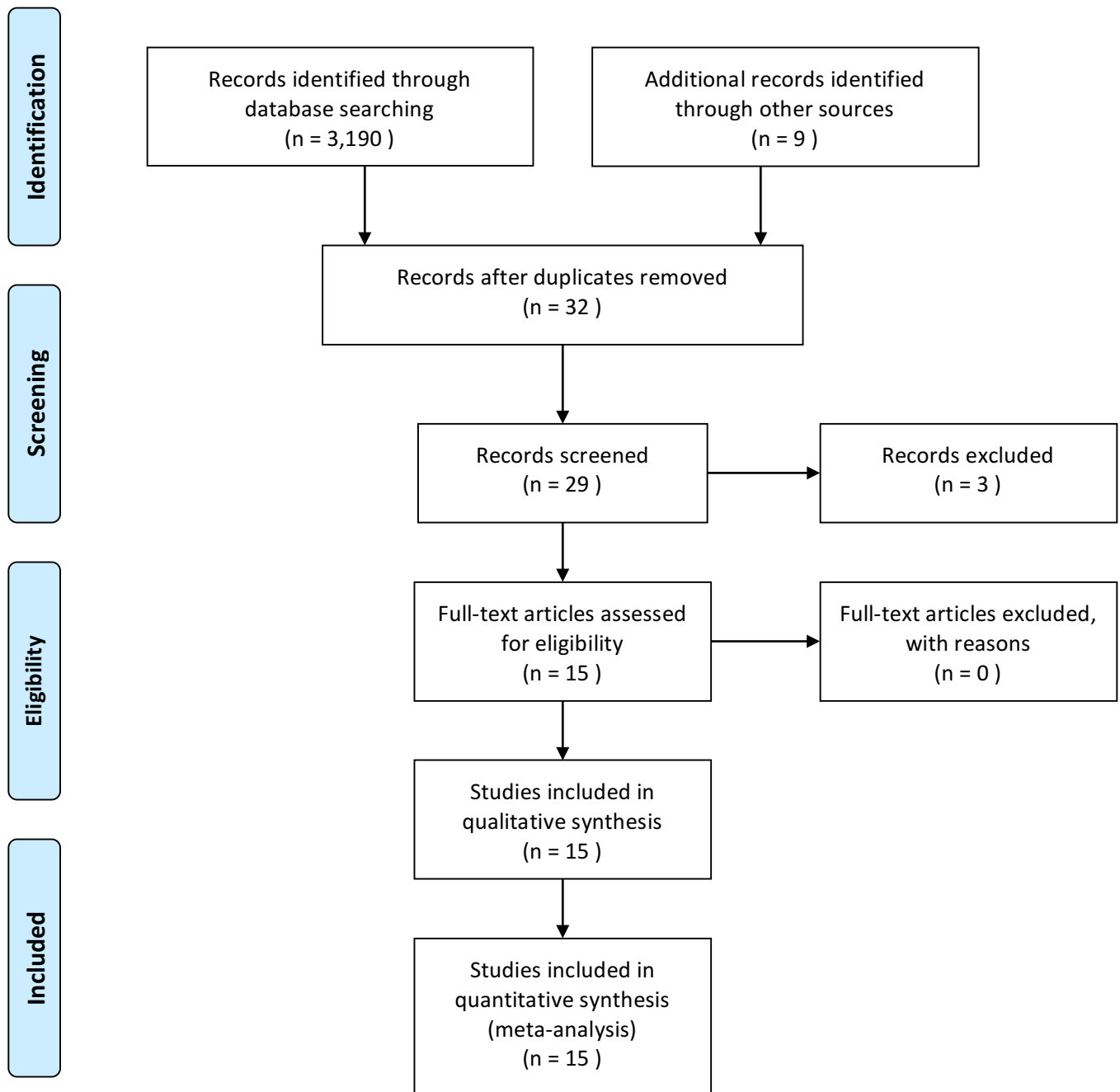

Supplement: S1 Fig — (PDF) [file pone.0187902.s001.pdf]
